# Lymph Node Metastases in Head and Neck Squamous Cell Carcinoma: The Association with Tumor Stage and Primary Tumor Location

**DOI:** 10.3390/jcm15114102

**Published:** 2026-05-26

**Authors:** Aldona Chloupek, Paweł Grab, Dariusz Jurkiewicz

**Affiliations:** Department of Otolaryngology and Laryngological Oncology with Clinical Department of Cranio-Maxillofacial Surgery, Military Institute of Medicine—National Research Institute in Warsaw, 04-141 Warsaw, Poland; pgrab@wim.mil.pl (P.G.); djurkiewicz@wim.mil.pl (D.J.)

**Keywords:** head and neck cancers, TNM staging, pathological, clinical

## Abstract

**Background**: Understanding lymph node involvement in head and neck cancers is crucial for developing effective treatment strategies and improving patient outcomes. Accurate identification of nodal metastases can enhance prognostic assessment, improve survival rates, and reduce the risk of recurrence. This study aimed to evaluate the association of lymph node metastases with primary tumor characteristics, with a particular focus on tumor stage and primary tumor location in head and neck squamous cell carcinoma (HNSCC). **Methods**: The study included 170 patients diagnosed with HNSCC at a single medical center between 2022 and 2025. Clinical and pathological assessments of the size and extent of primary tumors were performed according to the American Joint Committee on Cancer TNM classification, version 8. **Results**: The study cohort consisted of adult patients with a mean age of 61.9 years, of whom 40.6% were female. The tongue was the most common primary tumor site (54.7%), followed by the floor of the mouth (22.4%) and the jaw (8.8%). Clinical staging identified lymph node metastases (cN1 or higher) in 32.4% of patients, whereas pathological evaluation revealed nodal involvement in 38.9%. A statistically significant association was observed between tumor stage and the presence of lymph node metastases (*p* < 0.001). Additionally, the frequency of nodal metastases varied by anatomical site, with the highest rates observed in tumors of the floor of the mouth and the tongue. **Conclusions**: These findings suggest that both tumor stage and primary tumor location are associated with an increased risk of lymph node metastases. The results underscore the limitations of clinical staging in detecting nodal disease and highlight the prognostic significance of tumor stage and anatomical site in assessing metastatic risk.

## 1. Introduction

Head and neck cancer (HNC) represents a significant global health challenge, with over 800,000 new cases and more than 400,000 deaths reported annually [1]. Head and neck cancer comprises a heterogeneous group of malignancies affecting multiple anatomical sites, including the oral and nasal cavities, tongue, floor of the mouth, oropharynx, larynx, palate, tonsils, and salivary glands [1,2]. These malignancies arise from both mucosal and cutaneous epithelia, reflecting considerable biological complexity in their development and progression [3]. Squamous cell carcinoma is the predominant histological subtype, accounting for more than 90% of all HNC cases [4]. The biological heterogeneity of HNC is further reflected in its diverse etiology and progression patterns. Multiple factors, including tumor stage, primary tumor location, and the extent of lymph node metastases, influence prognosis. A substantial proportion of cases are diagnosed at advanced stages, largely due to inadequate recognition of early signs and symptoms [5,6].

Accurate staging is essential for prognostic stratification and the development of individualized treatment strategies [7,8]. In advanced disease, comprehensive treatment approaches—often involving surgery, radiation therapy, and chemotherapy—are typically implemented to ensure that patients receive appropriate, multimodal care. In contrast, when cancer is detected at an early stage, less intensive therapeutic strategies may be sufficient, thereby minimizing treatment-related morbidity while potentially improving overall outcomes [9,10].

The metastatic potential of HNC is influenced by tumor characteristic and the anatomical location of the primary lesion, reflecting heterogeneous lymphatic drainage patterns and complex interactions within the tumor microenvironment across different subsites [11,12,13]. The presence of lymph node metastases is a critical prognostic factor, significantly affecting both recurrence patterns and overall survival, with an associated reduction in survival of approximately 50% [14]. However, clinical detection of nodal metastases remains challenging due to the limited sensitivity of conventional diagnostic methods in identifying micrometastatic disease [15]. Consequently, patients classified as clinically node-negative may harbor occult metastases that are only detected upon pathological examination. Recent advances in molecular oncology have elucidated complex mechanisms underlying lymph node metastasis in HNC, including lymphangiogenesis, epithelial–mesenchymal transition, immune evasion, and interactions within the tumor microenvironment [16]. Emerging diagnostic technologies, such as liquid biopsy, multi-omics analyses, and targeted imaging probes, aim to improve early detection and enhance therapeutic precision [17].

This study aimed to analyze the association of lymph node metastases with two key variables: tumor (T) stage and anatomical location. By evaluating both clinical and pathomorphological staging parameters, this study sought to compare staging systems and explore potential discrepancies, as well as to address existing knowledge gaps regarding the complex interplay between tumor location and biological characteristics.

## 2. Materials and Methods

### 2.1. Data Collection

Medical records of patients with primary head and neck squamous cell carcinoma (HNSCC) were collected between 2022 and 2025 at the Military Institute of Medicine–National Research Institute in Warsaw, Poland. Clinical, pathological, and radiological data were extracted from electronic patient records within the institutional medical information system. Additional information on tumor histopathology was obtained from reports issued by the hospital’s pathology department. All patient data were anonymized prior to analysis. All procedures were conducted in accordance with the Declaration of Helsinki. An ethics approval was not required due to the retrospective study design.

### 2.2. Tumor Characteristics

Tumor histopathology was determined using standard diagnostic procedures performed at the time of initial diagnosis. The depth of invasion was assessed histopathologically. The presence of lymph node metastases was defined based on a combination of criteria, including histopathological examination, computed tomography evaluation, and clinical assessment by the treating physicians. the size and extent of the primary tumor were assessed according to the 8th edition of the American Joint Committee on Cancer tumor–node–metastasis (TNM) Classification (2017) [7]. The TNM system evaluates three key components: primary tumor characteristics (T), the extent of regional lymph node involvement (N), and the presence or absence of distant metastases (M). Clinical staging (cTNM) is based on physical examination and imaging modalities, including computed tomography, magnetic resonance imaging, and positron emission tomography. Pathological staging (pTNM) is based on the analysis of surgical specimens. In the 8th edition of the American Joint Committee on Cancer TNM classification the HPV status, depth of invasion and extranodal extension were added as criteria of tumor staging [7].

### 2.3. Statistical Analysis

Results were expressed as mean with standard deviation for continuous variables with a normal distribution and as median with interquartile range (median, IQR) for nonnormally distributed variables. Categorical variables were presented as percentages of the total study population. The Mann–Whitney U test was used for comparisons between two independent groups. For comparisons involving more than two groups, Kruskal–Wallis analysis of variance was applied, followed by post hoc Conover–Iman tests. The chi-squared test of independence was used for the analysis of categorical variables, with Yates’ correction or Fisher’s exact test applied when appropriate. A *p*-values of less than 0.05 was considered statistically significant. All analyses were performed using StatsDirect statistical software (v. 4.0.4).

## 3. Results

The study included 170 patients with HNSCC. The mean age was 61.9 ± 12.3 years, and women accounted for 40.6% of the cohort. The most common primary tumor site was the tongue (54.7%), followed by the floor of the mouth (22.4%) and the jaw (8.8%) (Table 1). The median tumor size was 28.5 mm (IQR, 18.0–47.0), with a mean size of 33.4 ± 19.1 mm. Clinically, most tumors were classified as cT2 (43.5%) or cT1 (27.1%), whereas pathological assessment revealed a more even distribution between pT1 (32.9%), pT2 (30.6%), and pT3 (22.4%). Most patients were clinically node-negative (cN0, 67.6%), while pathological examination confirmed pN0 status in 56.5% of cases. Detailed patient and tumor characteristics are presented in Table 1.

Based on clinical staging, lymph node metastases (cN1 or higher) were identified in 32.4% of patients, whereas pathological evaluation revealed nodal involvement in 38.9% of cases with available data. These findings suggest that clinical assessment alone may underestimate the presence of nodal disease. The overall frequency of lymph node metastases is summarized in Table 2.

An association between tumor size and the presence of lymph node metastases was observed for both clinical and pathological T staging (Table 3). Using clinical T classification, the frequency of clinically detected nodal metastases increased significantly from 8.7% in cT1 tumors to 35.1% in cT2, 45.8% in cT3, and 53.8% in cT4/T4a tumors (*p* = 0.0002). A similar trend was observed for pathologically confirmed nodal involvement, with rates increasing from 13.3% in cT1 to 77.3% in cT4/T4a tumors (*p* < 0.0001).

Consistent results were also obtained when tumor size was assessed pathologically (Table 3). The prevalence of clinically detected lymph node metastases increased progressively from 12.5% in T1 tumors to 66.7% in T4/T4a tumors (*p* < 0.0001). Pathological lymph node assessment similarly demonstrated a significant increase in the prevalence of nodal metastases with advancing tumor stage. Metastases were identified in 17.9% of patients with pT1 tumors, compared with 41.7% in pT2 and 41.9% in pT3 tumors, both significantly higher than in pT1 (*p* = 0.014 and *p* = 0.029, respectively). The highest frequency of nodal involvement was observed in pT4/pT4a tumors, where metastases were present in 81.8% of cases, which was significantly higher compared with pT1 (*p* < 0.0001), pT2 (*p* = 0.004), and pT3 (*p* = 0.009).

The frequency of lymph node metastases differed significantly according to the primary tumor location (Table 4). Clinically detected nodal metastases were most common in cancers of the floor of the mouth (50.0%) and the tongue (32.3%), whereas lower rates were observed for jaw (26.7%), lip (12.5%), skin (7.7%). No metastases were detected in tumors of the lateral pharyngeal wall. This difference was statistically significant (*p* = 0.033). Pathological assessment showed a similar pattern, with the highest rates of nodal involvement observed in tumors of the floor of the mouth (44.7%) and the tongue (40.9%). However, due to smaller subgroup sizes, these differences did not reach statistical significance (*p* = 0.408).

## 4. Discussion

Head and neck cancers represent a clinically challenging and biologically heterogeneous group of malignancies, requiring precise staging to guide optimal treatment and predict prognosis. Accurate assessment of lymph node involvement is critical, as nodal metastases are among the strongest predictors of survival and recurrence [14]. However, clinical staging may underestimate the extent of metastases, especially in early-stage tumors [18]. Additionally, tumor-related factors such as size and anatomical subsite play a major role in determining the risk of lymphatic spread [11,12,13].

The demographic profile of the studied population is consistent with global epidemiological data, according to which HNSCC predominantly affects older adults, with a median age of approximately 64 years [4]. Notably, recent evidence shows increasing incidence rates of HNC among younger patients, particularly in association with HPV infection [19,20]. The relatively high proportion of women in our cohort aligns with contemporary trends reporting a rising incidence of HNSCC among females [4]. The distribution of tumor locations (tongue cancers in 54.7%, floor of the mouth cancer in 22.4%, and jaw cancer in 8.8% of patients) is also comparable to previous studies, in which the tongue and floor of the mouth are the most common subsites within the oral cavity [21,22]. The oral cavity is particularly vulnerable to carcinogenesis due to its thin, often non-keratinized epithelium and rich lymphatic drainage, which may provide limited protection against carcinogenic exposure. Frequent contact with carcinogenic substances mixed with saliva, which tend to accumulate in dependent areas such as the floor of the mouth, results in prolonged exposure of these tissues to harmful agents, thereby facilitating both tumor initiation and early dissemination [23]. Human papillomavirus (HPV) infection has also emerged as an important risk factor for HNSCC. The mechanisms underlying HPV-related carcinogenesis in the head and neck region remain unclear. Research suggests that HPV may gain access to basal epithelial cells more readily in tonsillar crypts, where a unique microenvironment rich in immune cells may influence susceptibility to infection [24]. Furthermore, anatomical complexity and variations in local immune surveillance across different subsites may affect tissue responses to carcinogens and contribute to differences in tumor behavior [25].

In our cohort, clinical assessment identified lymph node metastases (cN1 or higher) in 32.4% of patients, whereas pathomorphological staging revealed nodal involvement in 38.9% of cases with available data. This discrepancy indicates that clinical staging underestimated nodal disease in a substantial proportion of patients. These findings are consistent with our previous research, which demonstrated that lymph node involvement assessed using cTNM staging was underestimated in 20.7% of patients with HNC [26]. Similar observations have been reported by Pinto et al., who found discordance between clinical and pathological N staging in up to 38% of cases, with approximately 25% of patients being upstaged postoperatively. Among patients initially classified as cN0, histopathological examination frequently reveals occult metastases, with reported rates ranging from 20% to 30%. This highlights the limitations of clinical examination and imaging in detecting microscopic nodal disease. Notably, these discrepancies were not associated with increased mortality or disease recurrence [18]. Punjabi et al. showed that concordance between clinical and pathological staging is generally higher at the extremes of disease (T1/N0 and T4/N3), whereas intermediate stages exhibit greater variability [27]. This discrepancy between cTNM and pTNM staging may be attributed to limitations in preoperative imaging as well as differences in diagnostic techniques [28]. Nevertheless, accurate staging remains essential for appropriate treatment selection, as it underpins clinical decision-making, risk stratification, and the choice of surgical and adjuvant therapeutic strategies.

A statistically significant association between tumor stage and lymph node metastases was observed in our study, regardless of whether clinical or pathological staging was applied. The prevalence of nodal involvement increased progressively from early-stage tumors (T1) to advanced tumors (T4/T4a). Pathological data demonstrated metastases in only 17.9% of pT1 tumors, compared with 81.8% in pT4/pT4a lesions. Walton et al. reported that tumor stage plays a crucial role in predicting the risk of lymph node metastases. As the T stage increases, the likelihood of nodal involvement rises steadily, peaking in T3–T4 disease, where the odds of lymph node metastases are more than doubled [29]. Our findings mirror this trend, confirming that increasing tumor stage substantially elevates the risk of regional metastases. Similarly, Ross et al. demonstrated a statistically significant correlation between T and N stages in HNSCC. Their study reported that neck metastases occurred in 29% of patients with cT1 tumors, whereas the incidence of lymph node involvement more than doubled in cT2 tumors, affecting 63% of patients. Additionally, the frequency of metastases increased across pathological T stages, reaching 36% in pT1, 44% in pT2, and 78% in pT3/4 tumors [30]. Of particular importance, tumor volume and thickness—key components influencing T classification—have also been identified as significant predictors of lymph node metastases. Patients with nodal involvement have been shown to present with significantly larger tumor volumes and greater tumor thickness compared with node-negative patients [30,31].

In the present study, the frequency of lymph node metastases varied significantly according to the anatomical site of the primary tumor. Clinically detected nodal metastases were most common in cancers of the floor of the mouth and the tongue, while markedly lower rates were observed in tumors of the skin, lip, and jaw. Although pathological differences did not reach statistical significance, likely due to smaller subgroup sizes, the same trend was observed. Ross et al. reported a similar pattern of metastatic distribution across subsites, with the highest rates observed in tumors of the posterior tongue (75%), followed by the floor of the mouth (45%) and the anterior tongue (33%) [30]. While some studies suggest that primary tumor location does not significantly influence the overall frequency of metastases [30,32], presented clinical data indicate that certain subsites are associated with a higher risk of nodal involvement. Furthermore, Van Leer et al. demonstrated that primary tumor location in HNSCC is associated with distinct patterns of lymph node metastases [33]. In a large analysis of surgically treated oral cavity squamous cell carcinoma, Carey et al. demonstrated that the rates of regional nodal disease as well as occult lymph node metastases varied between different subsites and tumor stages. The risk of occult disease was increased for higher pathologic T stage, positive margins, and LVI [22]. These findings suggest that, while primary tumor location may influence the anatomical pattern of lymphatic spread, nodal involvement is more strongly determined by tumor-related pathological features than by subsite alone.

The underestimation of nodal disease by clinical staging, combined with the strong association between tumor stage and metastatic risk, has important implications for treatment planning. Accurate pathological staging remains essential for optimizing adjuvant therapy and improving oncological outcomes.

Our study has several limitations. The retrospective design and incomplete pathological data for some patients may have introduced selection bias. Additionally, small sample sizes in certain anatomical subgroups limited the statistical power to detect significant differences in nodal metastases rates. Importantly, the HPV status of patients was not assessed, although the information about it could enable distinguishing two clinically different groups in terms of prognosis and nodal spread pattern [7].

## 5. Conclusions

In summary, our findings confirm that lymph node metastases in HNSCC are strongly associated with tumor stage and, to a lesser extent, primary tumor location. Clinical staging alone tends to underestimate nodal involvement, underscoring the importance of thorough pathological assessment. Tumors of the tongue and floor of the mouth, as well as advanced T-stage lesions, carry the highest risk of regional metastases. These findings support a risk-adapted approach to neck management and highlight the prognostic significance of accurate tumor staging in head and neck oncology.

## Figures and Tables

**Table 1:** Characteristics of the study population (n = 170).

Characteristics		Value
Age, mean ± SD		61.9 ± 12.3
Female sex		69 (40.6)
Tumor location		
	floor of the mouth	38 (22.4)
	jaw	15 (8.8)
	lateral pharyngeal wall	3 (1.8)
	skin	13 (7.6)
	lip	8 (4.7)
	tongue	93 (54.7)
Tumor size [mm]		
	mean ± SD	33.4 ± 19.1
	median (IQR)	28.5 (18.0–47.0)
Clinical T		
	cT1	46 (27.1)
	cT2	74 (43.5)
	cT3	24 (14.1)
	cT4	5 (2.9)
	cT4a	21 (12.4)
Clinical N		
	cN0	115 (67.6)
	cN1	25 (14.7)
	cN2	3 (1.8)
	cN2a	2 (1.2)
	cN2b	15 (8.8)
	cN2c	8 (4.7)
	cN3	2 (1.2)
Pathomorphological T		
	pT1	56 (32.9)
	pT2	52 (30.6)
	pT3	38 (22.4)
	pT4	4 (2.4)
	pT4a	20 (11.8)
Pathomorphological N		
	pN0	96 (56.5)
	pN1	22 (12.9)
	pN2a	2 (1.2)
	pN2b	15 (8.8)
	pN2c	6 (3.5)
	pN3	1 (0.6)
	pN3b	13 (7.6)
	pN3c	2 (1.2)
	pNx	13 (7.6)

Data are presented as number (%) of patients unless indicated otherwise. “cT1–T4a” and “pT1–T4a” denote the clinical and pathological assessment of the size and extent of the primary tumor according to the AJCC TNM classification ver.8 [7]; “cN0–N3” and “pN0–N3c” denote the clinical and pathological assessment of regional lymph node involvement according to the AJCC TNM classification ver.8 [7]; pNx denotes the number of pathological samples in which the nodal assessment was not possible. SD—standard deviation; IQR—interquartile range.

**Table 2:** Frequency of lymph node metastases based on clinical and pathological N staging.

Lymph Node Staging	N0	N1 and Higher
Clinical N stage	115/170 (67.6)	55/170 (32.4)
Pathological N stage	96/157 (61.1)	61/157 * (38.9)

Data are presented as number (%) of patients. * In the case of 13 samples the nodal assessment was not possible. “N” denotes the assessment of regional lymph node involvement according to the AJCC TNM classification ver.8 [7].

**Table 3:** Frequency of lymph node metastases depending on the clinical (cT) and pathological (pT) staging of the primary tumor.

Clinical T Stage	cT1	cT2	cT3	cT4 *	*p*-Value
Clinical lymph node metastases (cN1 and higher)	4 (8.7) n = 46	26 (35.1) n = 74 vs. cT1 (*p* = 0.002)	11 (45.8) n = 24 vs. cT1 (*p* = 0.001)	14 (53.8) n = 26 vs. cT1 (*p* < 0.001)	*p* = 0.0002
Pathological lymph node metastases (pN1 and higher)	6 (13.3) n = 45	29 (40.8) n = 71 vs. cT1 (*p* = 0.0015)	9 (47.4) n = 19 vs. cT1 (*p* = 0.006)	17 (77.3) n = 22 vs. cT1 (*p* < 0.0001) vs. cT2 (*p* = 0.006)	*p* < 0.0001
**Pathological T stage**	**pT1**	**pT2**	**pT3**	pT4 *	***p*** **-value**
Clinical lymph node metastases (cN1 and higher)	7 (12.5) n = 56	14 (26.9) n = 52	18 (47.4) n = 38 vs. pT1 (*p* = 0.0004)	16 (66.7) n = 24 vs. pT1 (*p* < 0.0001) vs. pT2 (*p* = 0.002)	*p* < 0.0001
Pathological lymph node metastases (pN1 and higher)	10 (17.9) n = 56	20 (41.7) n = 48 vs. T1 (*p* = 0.014)	13 (41.9) n = 31 vs. T1 (*p* = 0.029)	18 (81.8) n = 22 vs. T1 (*p* < 0.0001) vs. T2 (*p* = 0.004) vs. T3 (*p* = 0.009)	*p* < 0.0001

Data are presented as number (%) of patients. * cT4/pT4 stages include cT4+cT4a and pT4+pT4a, respectively; “cT0–cT4a” and “pT0–pT4a” denote the clinical and pathological assessment of the size and extent of the primary tumor according to the AJCC TNM classification ver.8 [7]; “N” denotes the assessment of regional lymph node involvement according to the AJCC TNM classification ver.8 [7]; n—number of patients by clinical and pathological stage.

**Table 4:** Frequency of lymph node metastases depending on primary tumor location.

Primary Tumor Location	Floor of the Mouth n = 38	Jaw n = 15	Lateral Pharyngeal Wall n = 3	Skin n = 13	Lip n = 8	Tongue n = 93	*p*-Value
Clinical lymph node metastases (cN1 and higher)	19 (50.0)	4 (26.7)	0 (0.0)	1 (7.7)	1 (12.5)	30 (32.3)	*p* = 0.033
Pathological lymph node metastases (pN1 and higher)	17 (44.7)	3 (27.3) (valid n = 11)	0 (0.0) (valid n = 0)	2 (28.6) (valid n = 7)	1 (12.5)	38 (40.9)	NS (*p* = 0.408)

Data are presented as number (%) of patients. “cN1” and “pN1” denote the clinical and pathological assessment of regional lymph node involvement according to the AJCC TNM classification ver.8 [7]; n—number of patients; NS—not significant.

## Data Availability

Data are available upon request from the corresponding author.

## Abbreviations

The following abbreviations are used in this manuscript:

AJCC	American Joint Committee on Cancer
cN/pN	Clinical/pathological assessment of regional lymph node involvement according to the AJCC TNM Classification (version 8)
cT/pT	Clinical/pathological assessment of the size and extent of the primary tumor according to the AJCC TNM Classification (version 8)
cTNM/pTNM	Clinical/pathological tumor–node–metastasis staging
CT	Computed tomography
HNC	Head and neck cancers
HNSCC	Head and neck squamous cell carcinoma
HPV	Human papillomavirus

## References

[1] Smith C.D.L., McMahon A.D., Purkayastha M., Creaney G., Clements K., Inman G.J., Bhatti L.A., Douglas C.M., Paterson C., Conway D.I. (2024). Head and Neck Cancer Incidence Is Rising but the Sociodemographic Profile Is Unchanging: A Population Epidemiological Study (2001–2020). BJC Rep..

[2] Bray F., Laversanne M., Sung H., Ferlay J., Siegel R.L., Soerjomataram I., Jemal A. (2024). Global Cancer Statistics 2022: GLOBOCAN Estimates of Incidence and Mortality Worldwide for 36 Cancers in 185 Countries. CA Cancer J. Clin..

[3] Stadler M.E., Patel M.R., Couch M.E., Hayes D.N. (2008). Molecular Biology of Head & Neck Cancer: Risks and Pathways. Hematol. Oncol. Clin. N. Am..

[4] Barsouk A., Aluru J.S., Rawla P., Saginala K., Barsouk A. (2023). Epidemiology, Risk Factors, and Prevention of Head and Neck Squamous Cell Carcinoma. Med. Sci..

[5] Panth N., Barnes J.M., Simpson M.C., Adjei Boakye E., Sethi R.K.V., Varvares M.A., Osazuwa-Peters N. (2020). Change in Stage of Presentation of Head and Neck Cancer in the United States before and after the Affordable Care Act. Cancer Epidemiol..

[6] Thompson-Harvey A., Yetukuri M., Hansen A.R., Simpson M.C., Adjei Boakye E., Varvares M.A., Osazuwa-Peters N. (2020). Rising Incidence of Late-Stage Head and Neck Cancer in the United States. Cancer.

[7] Amin M.B., Greene F.L., Edge S.B., Compton C.C., Gershenwald J.E., Brookland R.K., Meyer L., Gerss D.M., Byrd D.R., Winchester D.P. (2017). The Eighth Edition AJCC Cancer Staging Manual: Continuing to build a bridge from a population-based to a more "personalized" approach to cancer staging. CA Cancer J. Clin..

[8] Greenberg J.S., El Naggar A.K., Mo V., Roberts D., Myers J.N. (2003). Disparity in Pathologic and Clinical Lymph Node Staging in Oral Tongue Carcinoma. Cancer.

[9] Colevas A.D., Cmelak A.J., Pfister D.G., Spencer S., Adkins D., Birkeland A.C., Brizel D.M., Busse P.M., Caudell J.J., Durm G. (2025). NCCN Guidelines^®^ Insights: Head and Neck Cancers, Version 2.2025. J. Natl. Compr. Canc. Netw..

[10] Mordzińska-Rak A., Telejko I., Adamczuk G., Trombik T., Stepulak A., Błaszczak E. (2025). Advancing Head and Neck Cancer Therapies: From Conventional Treatments to Emerging Strategies. Biomedicines.

[11] Lengelé B., Hamoir M., Scalliet P., Grégoire V. (2007). Anatomical Bases for the Radiological Delineation of Lymph Node Areas. Major Collecting Trunks, Head and Neck. Radiother. Oncol..

[12] Iwanaga J., Lofton C., He P., Dumont A.S., Tubbs R.S. (2021). Lymphatic System of the Head and Neck. J. Craniofac. Surg..

[13] Mastronikolis N.S., Delides A., Kyrodimos E., Piperigkou Z., Spyropoulou D., Giotakis E., Tsiambas E., Karamanos N.K. (2024). Insights into Metastatic Roadmap of Head and Neck Cancer Squamous Cell Carcinoma Based on Clinical, Histopathological and Molecular Profiles. Mol. Biol. Rep..

[14] Xing Y., Zhang J., Lin H., Gold K.A., Sturgis E.M., Garden A.S., Lee J.J., William W.N. (2016). Relation between the Level of Lymph Node Metastasis and Survival in Locally Advanced Head and Neck Squamous Cell Carcinoma. Cancer.

[15] Kim S.-J., Pak K., Kim K. (2019). Diagnostic Accuracy of F-18 FDG PET or PET/CT for Detection of Lymph Node Metastasis in Clinically Node Negative Head and Neck Cancer Patients; A Systematic Review and Meta-Analysis. Am. J. Otolaryngol..

[16] Constantin M., Chifiriuc M.C., Bleotu C., Vrancianu C.O., Cristian R.-E., Bertesteanu S.V., Grigore R., Bertesteanu G. (2024). Molecular Pathways and Targeted Therapies in Head and Neck Cancers Pathogenesis. Front. Oncol..

[17] Sattar T., Nazir I., Jabbar M., Malik J., Afzal S., Hanif S., Mosaddad S.A., Hussain A., Tebyaniyan H. (2025). Current Innovations in Head and Neck Cancer: From Diagnostics to Therapeutics. Oncol. Res..

[18] Pinto J.V., Sousa M.M., Silveira H., Vales F., Moura C.P. (2023). Comparison of Clinical and Pathological Staging in Patients with Head and Neck Cancer After Neck Dissection. Int. Arch. Otorhinolaryngol..

[19] Wierzbicka M., Pietruszewska W., Maciejczyk A., Markowski J. (2025). Trends in Incidence and Mortality of Head and Neck Cancer Subsites Among Elderly Patients: A Population-Based Analysis. Cancers.

[20] Hsieh R.W., Gooding W.E., Nilsen M., Kubik M., Kelly Z., Sridharan S., Skinner H., Iheagwara U., Zevallos J.P., Duvvuri U. (2025). Association of Patient and Tumor Characteristics with Outcomes in Young Head and Neck Squamous Cell Carcinoma Patients. Clin. Otolaryngol..

[21] Meccariello G., Cammaroto G., Ofo E., Calpona S., Parisi E., D’Agostino G., Gobbi R., Firinu E., Bellini C., De Vito A. (2019). The Emerging Role of Trans-Oral Robotic Surgery for the Detection of the Primary Tumour Site in Patients with Head-Neck Unknown Primary Cancers: A Meta-Analysis. Auris Nasus Larynx.

[22] Carey R.M., Anagnos V.J., Prasad A., Sangal N.R., Rajasekaran K., Shanti R.M., Cannady S.B., Newman J.G., Brant J.A., Brody R.M. (2023). Nodal Metastasis in Surgically Treated Oral Cavity Squamous Cell Carcinoma. ORL J. Otorhinolaryngol. Relat. Spec..

[23] Neville B.W., Day T.A. (2002). Oral Cancer and Precancerous Lesions. CA A Cancer J. Clin..

[24] Golusiński W., Golusińska-Kardach E., Machczyński P., Szewczyk M. (2025). HPV-Driven Head and Neck Cancer: The European Perspective. Viruses.

[25] Han N., Zhou D., Ruan M., Yan M., Zhang C. (2023). Cancer Cell-Derived Extracellular Vesicles Drive Pre-Metastatic Niche Formation of Lymph Node via IFNGR1/JAK1/STAT1-Activated-PD-L1 Expression on FRCs in Head and Neck Cancer. Oral Oncol..

[26] Chloupek A., Kania J., Jurkiewicz D. (2023). Concordance between Clinical and Pathological T and N Stages in Polish Patients with Head and Neck Cancers. Diagnostics.

[27] Punjabi N., Hondorp B., Macias D., Liu Y., Inman J. (2024). Patterns of Discordance Between Clinical and Pathologic Stage in Head and Neck Squamous Cell Carcinoma. Int. J. Radiat. Oncol. Biol. Phys..

[28] López F., Mäkitie A., de Bree R., Franchi A., de Graaf P., Hernández-Prera J.C., Strojan P., Zidar N., Strojan Fležar M., Rodrigo J.P. (2021). Qualitative and Quantitative Diagnosis in Head and Neck Cancer. Diagnostics.

[29] Walton E., Cramer J.D. (2020). Predictors of Occult Lymph Node Metastases in Lip Cancer. Am. J. Otolaryngol..

[30] Ross G.L., Soutar D.S., MacDonald D.G., Shoaib T., Camilleri I.G., Robertson A.G. (2004). Improved Staging of Cervical Metastases in Clinically Node-Negative Patients with Head and Neck Squamous Cell Carcinoma. Ann. Surg. Oncol..

[31] Jang J.Y., Kim M.J., Ryu G., Choi N., Ko Y.-H., Jeong H.-S. (2015). Prediction of Lymph Node Metastasis by Tumor Dimension Versus Tumor Biological Properties in Head and Neck Squamous Cell Carcinomas. Cancer Res. Treat..

[32] Yu Y.-F., Cao L.-M., Li Z.-Z., Zhong N.-N., Wang G.-R., Xiao Y., Wu Q.-J., Liu B., Bu L.-L. (2024). Frequency of Lymph Node Metastases at Different Neck Levels in Patients with Oral Squamous Cell Carcinoma: A Systematic Review and Meta-Analysis. Int. J. Surg..

[33] van Leer B., Leus A.J.G., van Dijk B.A.C., van Kester M.S., Halmos G.B., Diercks G.F.H., van der Vegt B., Vister J., Rácz E., Plaat B.E.C. (2022). The Effect of Tumor Characteristics and Location on the Extent of Lymph Node Metastases of Head and Neck Cutaneous Squamous Cell Carcinoma. Front. Oncol..

